# Prolonged patients’ In-Hospital Waiting Period after discharge eligibility is associated with increased risk of infection, morbidity and mortality: a retrospective cohort analysis

**DOI:** 10.1186/s12913-015-0929-6

**Published:** 2015-06-25

**Authors:** Maya Rosman, Orna Rachminov, Omer Segal, Gad Segal

**Affiliations:** Department of internal medicine “T’, Chaim Sheba Medical Center@, Ramat Gan, Israel; Department of Nursing Management, Chaim Sheba Medical Center@, Ramat Gan, Israel; Sackler faculty of Medicine, Tel-Aviv University, Tel-Aviv, Israel

**Keywords:** Nosocomial infection, Hospital acquired infection, Hospital stay, In-hospital mortality, Pneumonia, Urinary tract infection, Sepsis

## Abstract

**Background:**

Prolonged, inappropriate hospital stay after patients’ eligibility for discharge from internal medicine departments is a world-wide health-care systems’ problem. Nevertheless, the extent to which such surplus hospital stays are associated with infectious complications, their time frame of appearance and their long-term implications was not previously addressed.

**Methods:**

We conducted a retrospective cohort analysis of patients experiencing an In-hospital Waiting Period (IHWP) after discharge eligibility in a single, tertiary hospital.

**Results:**

We screened the records of 245 patients out of which 104 patients fulfilled our inclusion criteria. The mean length of IHWP was 15.7 ± 4.79 day during which 9(8.7 %) patients died. The study primary composite end-point, in-hospital mortality or hospital acquired infection (pneumonia, UTI or sepsis) occurred in 32(31 %) patients. The most hazardous time was during the first 3 IHWP days: 63.7 % of patients experienced a complication and 44 % of the total complications occurred during this period. The occurrence of any complication during IHWP was associated, with statistical significance, with increased risk of mortality during the first year after IHWP initiation (HR = 6.02, *p* = 0.014).

**Conclusion:**

Prolongation of hospital stay after patients are deemed to be discharged from internal medicine departments is associated with increased morbidity and mortality, mainly during the first surplus days of in-hospital stay. Efforts should be made to shorten such hospital stays as much as possible.

## Background

There is a large body of evidence supporting the fact that prolonged hospitalizations are associated with increased risk for in-hospital complications including infections and deep venous thrombosis [1, 2]. However, most of the published data deal with pre-specified populations and the prolonged hospital stay is considered as an outcome rather than a causative factor for complications (e.g., after a certain surgical procedure like orthopedic surgery [3] or a coronary artery bypass graft surgery [4]). Only few researchers addressed this issue with relation to the general, most often frail and elderly population admitted to internal medicine departments. Many of these patients experience unnecessary, prolonged hospitalization periods while waiting for a suitable nursing or rehabilitation facility.

In the current study we addressed the issue of surplus infectious complications potentially resulting from prolonged, inappropriate hospital stay, for patients that were defined as eligible for hospital discharge from internal medicine departments, either for rehabilitation or a nursing home. The reasons for prolonged hospital stay were mainly low availability of rehabilitation and nursing beds in the aforementioned facilities. We did not include patients that were mechanically ventilated or patients found to be carriers of Carbapenemase resistant bacteria, both populations deemed to experience prolonged hospital stays that are especially difficult to shorten. Our patient population comprised mainly of patients diagnosed of suffering from stroke and patients recovering from severe, acute illness. These two groups of patients are routinely checked by a specialist in geriatric medicine who recommended a rehabilitation period or functional deconditioning, respectively.

## Methods

After approval of an institutional review board, the ethics committee of the Sheba medical center, patients’ medical records were analyzed by a single investigator. Study patients were all consecutive patients from internal medicine departments in our hospital. As such, the study population is considered representative of our general population of patients admitted to internal medicine departments. The following items were collected: patients’ demographics (e.g., age and gender); clinical background (Charlson Comorbidity Index (CCI), age corrected) [5] and records of the following infectious complications diagnosed during their in-hospital waiting period: pneumonia, urinary tract infection (UTI), sepsis, clostridium difficile associated colitis and death. The primary composite end-point of the study was the occurrence of in-hospital mortality or hospital acquired infection (pneumonia, UTI or sepsis). The risk for separate complications as well as the risk for the primary composite endpoint was calculated per each day during the IHWP. In light of the results of analyses, with consideration of IHWP on a day-by-day level, and in order to achieve a better understanding of the effects mentioned above, it was concluded that IHWP days should be divided according to time periods as follows: the first three days of the IHWP, days 4 to 7 of IHWP and the 8^th^ day and forth of the IHWP. In-hospital mortality was determined according to the patients’ medical record while long-term; post discharge mortality data were obtained from the national population registry.

### Statistical analysis

Variables were expressed as mean ± SE, and categorical data were summarized as frequencies and percentages. The clinical characteristics of the patients at baseline were compared between the subgroups, with the use of the chi-square test for dependency of dichotomous variables. The Binary Logistic Regression model was used for testing the effect of IHWP length on occurrence of the primary complications, including patients’ mortality. The Kaplan–Meier method was used to determine cumulative probabilities of the occurrence of the various complications in patients, including patients’ mortality. The Hierarchical Binary Logistic Regression model was used to estimate the effect of IHWP length on the occurrence of various complications in patients, under the confounder variable CCI (age adjusted).

## Results

The study was conducted in a tertiary hospital. Electronic medical records (EMR) of 245 consecutive patients that were eligible for discharge from internal medicine departments during the period between 1^st^ of January to the 30^th^ of June 2013 and stayed for an in-hospital waiting period were initially screened. Patients’ demographic characteristics are detailed in Table 1.

**Table 1:** Patients’ baseline demographic characteristics

Patients’ characteristics	Value
Age (years, mean ± SE)	76 ± 11
Male gender (%)	52.9 %
Charlson Comorbidity Index (age adjusted, mean ± SE)	6.83 ± 2.8
Norton score (mean ± SE)	11.9 ± 2.52
Length of hospital stay (days prior to IHWP, mean ± SE)	14.1 ± 2.83
Length of IHWP (days, mean ± SE)	15.7 ± 4.8

**Fig. 1:**
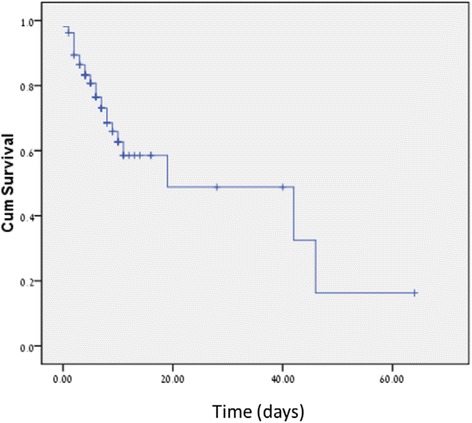
Un-adjusted risk of the primary composite endpoint (occurrence of in-hospital mortality or hospital acquired infection) during the IHWP

The in-hospital waiting period (IHWP) was, in most cases, a result of their need for a follow-up stay in a chronic medical facility (geriatric rehabilitation department, chronic hospitalization due to on-going chronic diseases etc.). Only 104 patients’ records that fulfilled the inclusion criteria and had sufficient data were included in our final analysis. The mean length of IHWP was 15.7 ± 4.79 days. A total of 9(8.7 %) patients died during IHWP. The primary composite end-point occurred in 32(31 %) patients. 9(8.7 %) patients suffered from pneumonia, 14(13 %) had UTI, 9(8.7 %) had sepsis and 1(0.96 %) patient was infected with clostridium difficile during the IHWP. The mean duration to occurrence of any complication was 6.2 days (o to 46 days). The mean length of IHWP of patients who died during this period was 10 days (2 to 42 days). Figure 1 describes the un-adjusted risk of the primary composite endpoint (occurrence of in-hospital mortality or hospital acquired infection) during the IHWP.

**Fig. 2:**
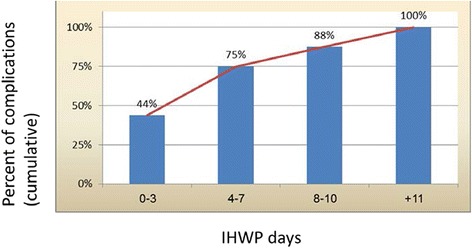
Cumulative burden of any complications along the whole length of IHWP

The risk of death during the first 3 days of IHWP was the greatest (22.72 %, *p* = 0.05) and was significantly greater than the risk of death during days 4 to 7 (2.04 %, *p* = NS) and the risk of death at day 8 and beyond (9.09 %, *p* = NS). The risk of experiencing the study’s primary composite end-point (in-hospital mortality or hospital acquired infection was at its height at IHWP initiation: the risk during the first 3 days of IHWP was 63.7 % (*p* = 0.005). The risk reduced to 20.44 % (*p* = NS) at days 4 to 7. On day 8 and beyond to IHWP, the risk of in-hospital mortality or hospital acquired infection was 24.25 % until the end of the IHWP. 44 % of all complications occurred during the first 3 days of IHWP, 31 % during days 4 to 7, 12 % during days 10 to 12 and 13 % after day 11. As a whole, the results can evidently imply, that the cumulative burden of complications increases as the IHWP lengthens (Fig. 2). The differences between the sections of the IHWP, with regard to the risk of the study’s main outcome, were not affected after adjusting the relative risk for the possible confounding effect of the Charlson Comorbidity Index (CCI age adjusted).

The occurrence of any complication during IHWP was associated, with statistical significance, with increased risk of mortality during the first year from IHWP initiation (HR = 6.02, *p* = 0.014).

## Discussion

During daily rounds in internal medicine departments, it is a custom to encourage, both patients and families, to go home as soon as it is considered appropriate. However, many patients are staying in-hospital after discharge eligibility: some due to family incompetence or failure in reassurance by the attending physician, some due to objective obstacles in assuring appropriate treatment continuity in the setting of primary medicine and some due to lack of vacant beds at their next, rehabilitation or nursing facility. We wanted to make sure, that our stringent policy of advancing discharge due to fear of nosocomial infections is indeed justified. We did not find in the relevant literature, previous studies that fully answered our needs with regard to the population of elderly patients hospitalized in internal medicine departments. Harkanen and co. showed that prolonged hospital stay was associated with increased risk for adverse drug reactions [6]. In another study [7], prolonged hospitalization was associated with increased in-hospital mortality. Majeed and co. [8] showed that especially the elderly population is subject to increased vulnerability and subsequent high frequency of nosocomial complications from prolonged hospital stays. D’illo and co. in a study from Italy showed that 55 % of patients, eligible for discharge after acute hospitalization, were discharged later than expected due to low availability of rehabilitation resources [9]. A total of 4505 inappropriate hospitalization days were documented for a cohort of 1083 individuals only. New and co. [10], describing the “patient flow as a major problem in hospitals”, appreciated that overall, 12 % of the length of the acute hospital stay, amongst 360 patients they included in their study, were surplus length of stay after these patients were deemed ready for transfer for rehabilitation. The problem of patient flow and its impact on hospital stay is significant in the United States also, as reflected in a review by Kane [11], describing the transfer from hospital to post-hospital care as the “big leap”. In a retrospective observational pilot study, Foer et al. [12] found that non-medical factors accounted for nearly one third of all “long-stay” hospitalizations. They also found out delays in finding a placement in a nursing facility to be the most common reason for such stays. Indeed, the best timing for hospital discharge after a stay in the internal medicine department is largely unknown. Many studies showed and healthcare professionals agree that this should be as soon as possible, for the vast majority of patients. Previous publications address relevant issues that are aimed at different end-points, such as diminishing re-admission rates [13, 14].

## Conclusions

The main finding of our study is that, whatever was the cause for discharge delay, inappropriate in-hospital stay is associated with increased risk of infection and both short- and long-term mortality. We found that the most hazardous time is during the first three days after discharge is deemed appropriate. We can only speculate why the risk is reduced thereafter: it could be that the medical attention is significantly reduced during the first days, maybe too soon. Also, it is possible that during the following days, after 4 days of surplus hospital stay, reduced medical attention could even harbor advantages for the surviving patients: reduced intensity of invasive procedures and medication changes could promote stabilization on behalf of the recovering patient. It is plausible to think that after a certain period, the patient becomes more of a tenant, less inflicted by nosocomial threats.

Nevertheless, our findings show that short-term complications, during the initiation of surplus hospital stay should not be overlooked, even if their occurrence is diminishing, since these complications are significantly associated with increased risk of long-term mortality.

### Study limitations

Our study has several limitations: it is a retrospective study and therefore, no causality could be inferred from its results. Furthermore, prospective studies are warranted in order to affirm these findings although it would not be easy to design a study in which a certain group of patients would be randomized to stay in-hospital after deemed eligible for discharge. A potential limitation is the fact that there could be geographical alterations in the causes for prolonged, inappropriate in-hospital waiting. Such diversity could potentially affect our results.
